# Ninety years of convulsive therapy: Ugo Cerletti’s pioneering role in advancing safe and effective treatment for severe psychiatric illnesses

**DOI:** 10.47626/2237-6089-2024-0830

**Published:** 2025-09-18

**Authors:** Larissa Junkes, Antonio E. Nardi

**Affiliations:** 1 Instituto de Psiquiatria Universidade Federal do Rio de Janeiro Rio de Janeiro RJ Brazil Instituto de Psiquiatria, Universidade Federal do Rio de Janeiro, Rio de Janeiro, RJ, Brazil.

**Figure 1:**
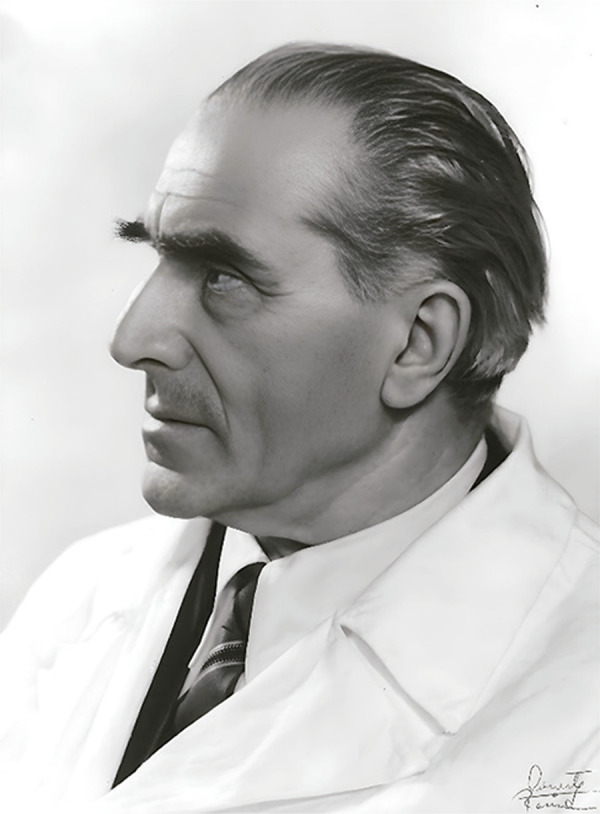
Ugo Cerletti, an Italian psychiatrist, was the first scientist to propose the use of electricity for inducing therapeutic seizures.

In the vast narrative of scientific discovery, few topics have sparked as much debate as the use of convulsive therapy in psychiatry. It was 90 years ago, in 1934, that the initial experiments with insulin shock by Manfred Sakel, in Vienna, and camphor injection by Ladislas J. Meduna, in Budapest, were carried out. These early endeavors sought to induce generalized convulsive seizures as a means to alleviate psychotic symptoms in a patient suffering from schizophrenia, and in another patient with catatonic stupor, respectively. Expanding on these foundational ideas, Italian psychiatrist Ugo Cerletti (1877-1963) and his disciple Lucio Bini explored the potential of using electricity to specifically induce convulsive seizures in the brain, thus circumventing the risks associated with passing electrical current through the heart. Cerletti^1^ (Figure 1) emerged as the first to propose the use of electricity for seizure induction, aiming for therapeutic outcomes akin to those obtained with cardiazol and insulin therapies.

In addition to being acclaimed for his contributions to medical science, Ugo Cerletti was a multifaceted scientist and researcher, possessing a broad spectrum of interests that extended far beyond the confines of psychiatry. Prior to his experiments with electroconvulsive therapy (ECT), he dedicated three decades to the study of neuropathology, with a particular emphasis on neuroglia. His scholarly pursuits were diverse, encompassing a wide array of medical disciplines including neuroanatomy, as well as the study of goiter, cretinism, and congenital syphilis.^2^ Cerletti’s ingenuity was not limited to the realm of medicine. During World War I, he proposed an innovative strategy for enhancing the camouflage of army troops traversing the Alps by suggesting they don white uniforms to blend seamlessly with the snowy landscape. Additionally, he invented artillery missiles with delayed-action fuses. These were utilized by the Italian and French armies to create minefields in enemy territories.^3^

Following his foundational training in medicine, with specializations in neurology and neuropsychiatry, Ugo Cerletti further enriched his expertise abroad, studying under Pierre Marie and Ernest Dupré in Paris. His educational journey then extended to Germany, where he learned from eminent figures such as Emil Kraepelin, Aloysius Alzheimer, and Franz Nissl. Upon returning to Rome, Cerletti embarked on pioneering research into ECT at La Sapienza University, focusing on its application for treating psychiatric illnesses in humans. In collaboration with Lucio Bini, who had developed an early prototype of an ECT device, they administered the first ECT sessions in April 1938 to a patient with schizophrenia, who exhibited severe psychiatric symptoms, including delusional thoughts, hallucinations, and mannerisms.^2^ Remarkably, the treatment led to a significant improvement in the patient’s condition, demonstrating ECT’s potential as a viable treatment option for psychiatric disorders that lacked effective therapeutic solutions at the time.

In 1940, after meticulous research, Cerletti and his team finally published a special edition in the *Rivista Sperimentale di Freniatria* detailing the promising, effective, and cost-efficient therapy that they devised. The two-year interval between the application of this therapy and its publication was a deliberate choice by Cerletti, who deemed it prudent to delay public disclosure until a thorough understanding and analysis of the most suitable method for its application were achieved.^4^ This period of cautious evaluation was also influenced by the broader historical context, as Europe, including Italy, was under the grip of totalitarian regimes. In his address at the XXIII Congress of the Italian Society of Psychiatry in 1946, the first congress after the war, Cerletti openly criticized the “non-scientific use” of recent therapeutic discoveries in psychiatry, highlighting his commitment to scientific integrity and ethical practice.^4^

Electroconvulsive therapy, recognized for its remarkable efficacy and simplicity in administration, transcended its initial therapeutic boundaries, finding applications that strayed far from its intended use, including as a means of punishment and for maintaining patient custody. This divergence marked the onset of what came to be known as the “Era of Electroshock,” a period particularly notable in historical psychiatric practices. Such practices were not uncommon in the psychiatric hospitals of the past, and the repercussions of these applications have left a lasting stigma on ECT, especially in countries like Italy – Cerletti’s homeland – and Brazil. In Brazil, there are still vivid recollections of ECT being misused as a tool for torture and behavioral control on individuals without mental disorders, contributing to a societal stigma that leads to its underutilization. Despite these historical misapplications, it is critical to dissociate ECT from its past ideological misuses and recognize its value as a pragmatic medical intervention with specific clinical indications. ECT remains a recommended treatment for patients suffering from severe conditions such as treatment-resistant depression, schizophrenia characterized by acute psychotic episodes, catatonia and resistance to clozapine, acute mania, severe mixed affective states, and for those who are at a high risk of suicide. The acceptance and application of ECT vary globally, with countries like the United States, Australia, New Zealand, and several European nations exhibiting a broader acceptance and utilization of the procedure.^5^ This broader acceptance may be attributed to the structure of mental health services, the accumulation of professional experience, and the strong confidence in its safety and efficacy. In contrast, in Brazil, the media often exacerbates the negative perception of ECT, fueling public opposition against its use. This opposition persists despite the procedure not only being safe and effective but also the best treatment option available for certain cases.

Scientist John Ziman, who pondered the intricate relationship between science and society, offers a reflection that deeply resonates within the ethical considerations of medical practices: “Ethics is not just an abstract intellectual discipline. It is about the conflicts that arise in trying to meet real human needs and values.” This insight is relevant in the ongoing debate surrounding the use of ECT, where clear-cut distinctions between right and wrong are challenging to delineate, and the dialogue is enriched by a diversity of valid perspectives. On one side of this debate, there is a strong ethical stance against the indiscriminate use of ECT, especially when applied without consent or utilized for punitive reasons. On the other side, the argument in favor of ECT emphasizes its efficacy and the vital role it can play in the treatment landscape for psychiatric conditions that are resistant to other forms of therapy. This position underscores the necessity of ensuring that patients have access to the most effective treatment available. This situation calls for a balanced approach, where we consider the needs and concerns of the patient in order to find a careful solution that addresses the underlying issues without disregarding important ethical considerations.

Science is constantly evolving and developing, offering no definitive answers but rather the best approach based on current knowledge. In the realm of ECT, the incidence of muscle damage, bone fractures, and dental injuries, which were once common, has significantly decreased. The procedure today involves safety measures, including the use of anesthesia and muscle relaxants, the application of brief and ultra-brief electrical pulses, and the employment of an electroencephalogram to record brain activity. Constant monitoring and commitment to patient safety are integral to the process. Just as with any form of treatment, whether medical or surgical, the potential risks and benefits of ECT must be weighed before it is recommended.

Newer forms of neuromodulation therapy, more recent than ECT, such as transcranial magnetic stimulation (TMS), photobiomodulation (PBM), transcranial ultrasound stimulation (TUS), deep brain stimulation (DBS), and vagus nerve stimulation (VNS), are currently under research as potential treatments. These methods aim to decrease neuroinflammation and reduce the release of inflammatory factors, thereby stabilizing mood.^6^ However, to date, none of these therapies have been proven to be superior to ECT. Similarly, even newer drugs, including ketamine, have not demonstrated superiority over ECT as a therapeutic alternative for treatment-resistant depression.^7^ According to the guidelines of reputable universities and both national and international medical associations, ECT remains a fundamental treatment option for mood disorders that are resistant to other treatments, as well as for other serious illnesses.^5^

Ugo Cerletti, with determination and perseverance, revolutionized the theoretical and therapeutic approaches of his era by successfully integrating safety and effectiveness in convulsive therapy. He was the pioneer who introduced the concept of using electricity in the treatment of mental illnesses, conducting tests and validating its application. His innovative spirit and significant contributions have left a lasting impact on modern psychiatry, garnering him widespread recognition both within Italy and on an international scale. In recognition of his contributions, Cerletti was appointed president of the Italian Psychiatric Association and Honorary President of the Italian Society of Neurology. He was honored with awards from the Academy of Italy and was a nominee for the Nobel Prize. Cerletti also received honorary titles from universities across the globe, including those in Rio de Janeiro, São Paulo, Paris, and Montreal. Furthermore, he was acknowledged by prestigious institutions such as the American Neurological Association and the American Society for Medical Psychiatry. His commitment to advancing his innovative ideas, along with his advocacy for ethical practices in psychiatry, continues to serve as a source of inspiration for many in the field.
